# Progress in Roentgen Ray Work

**Published:** 1902-01-04

**Authors:** 


### PROGRESS IN ROENTGEN RAY WORK.

**Diagnosis.**—Of the value of the X-ray fresh testimony is constantly forthcoming. Rudis-Jicinsky[1] reports a case of gall-stones where the skiagram showed two stones, the diagnosis being verified at the subsequent operation. Also a case of obscure head mischief, where the X-ray revealed under the parietal bone at the sagittal suture a large epidural clot. The clot, amounting to four ounces, was removed, and recovery followed in three weeks without any complication. In cases of renal calculus J. Hutchinson, junr.,[2] considers it most important that an experienced and expert radiographer should be called in, because (1) The X-rays (except, perhaps, in stout subjects or in the case of very small stones) enable an exact diagnosis as to size, position, and number of renal calculi to be made; and (2) They enable the surgeon in performing the operation of nephrolithotomy to do so with the least possible injury to the kidney. He makes one exception to their value, viz. in very fat subjects, in which cases, he states, the rays are valueless. A very interesting case is reported by Barclay Baron[3] of a large aortic aneurism involving the whole of the aortic arch, the greatest enlargement being on the right side. The only symptom pointing to this lesion was paralysis of the right vocal cord, there being practically no physical signs in the thorax. In such cases as this the fluoroscope is most valuable, as by its use the pulsation of the aorta can be seen most clearly; but, in surgical cases, Johnson[4] truly remarks that it has proved rather unsatisfactory and disappointing. C. L. Leonard[5] discussing what reliance can be placed upon the image produced by the X-ray from a medico-legal standpoint, says that the value and accuracy of the X-ray for medico-legal purposes are entirely dependent upon the skill of the expert using this method, and that neither lawyer, photographer, nor professional witness without special experience is capable of correctly interpreting a skiagraph, nor is he competent to give testimony thereon. Miles[6] also draws attention to the fallacies in the interpretation of skiagrams of fractures, and quotes several instances where error is very likely to occur. This author also protests against the "tendency to make the ordinary signs and symptoms of fractures and dislocations subsidiary to the evidence afforded by radiographic plates." Again, "the teacher of surgery should remember that the majority of his pupils will practise their profession under conditions in which a routine appeal to the X-rays will be impossible. The student, therefore, should be taught to rely chiefly on the ordinary diagnostic means."

**As a Therapeutic Agent.**—Sjögren and Sederholm,[7] of Stockholm, give the result of X-ray treatment in a series of cases:—*Lupus Vulgaris.*—In

27 cases expectations were not quite realised. "The treatment is ideal, in that it is free from pain and other inconveniences (unless reaction is carried too far), and that the new skin, though thin and atrophic, is not scar tissue. It does not contract, like that which is formed by other methods of treatment." Moreover the chronic dermatitis, or eczema, which is always present in severe lupus, soon disappears. But the lupus tubercles themselves offer an obstinate resistance. Sjögren and Sederholm have found it of great advantage to treat them individually by galvano-puncture or other suitable measures while the Roentgen treatment is being carried on. Recurrences are met with in about the same proportion as after other methods of treatment. *Lupus Erythematosus.*—The conclusion arrived at by the authors after treating six cases is that "if the Roentgen treatment is kept up sufficiently long it appears to be successful." *Chronic Eczema.*—In 10 cases the result was good in all, and in the greater number the cure was complete. *Chronic Ulcers.*—Four cases. Cause of ulceration uncertain, the only point in common between them being that they had resisted other forms of treatment. Two cases were cured and one greatly benefited. *Epithelioma.* — Five cases. The effect on rodent ulcer is conspicuously good. In some of the cases the ulcer healed without reaction having been excited. *Warts.*—The effect was excellent in four cases. In one case there were upwards of 200 warts on one hand. All the small ones had disappeared after fifteen sittings and the larger ones had very much diminished in size. A contribution to the pathology of Raynaud's disease has been made by means of the X-rays. Carl Beck[8] took careful skiagrams of two cases, which showed an osseous proliferation at the upper end of the second phalanges, and the epiphyseal ends of the second, third, and fourth metacarpal bones were thickened. These cases go to prove that the nutrition of the bone is apt to suffer in this disease.

**Localisation of Foreign Bodies.** — In military surgery accurate localisation of bullets in the body is an absolute necessity, for as Edwards[9] points out with the high-velocity firearms recently introduced, a bullet is situated so far from the entrance wound that no probe is capable of reaching it. Throughout his service in South Africa he employed the usual "crossed-thread" method, and in no single case was a bullet missed after a localisation had been made. Fox[10] describes a method of localising a bullet in the brain by means of two wires, one encircling the skull and another carried over the vertex at right angles to the first. A skiagram is taken of the skull with the wires on it, and the measurements are then easily made. *Apparatus.*—Batten[11] has invented a rectifier for transforming alternating currents into direct ones, which bids fair to be very useful to workers who only have the alternating current supply at their command, either for charging accumulators or for working the coil.

[1] Annals of Surgery, June, 1901. [2] Brit. Med. Jour., Oct. 19, 1901. [3] Brit. Med. Chir. Jour., Sep., 1901. [4] Med. Record, Sep., 1901. [5] Med. Record, July 27, 1901. [6] Scottish Med. and Surg., Sep., 1901. [7] Fortschritte auf dem Gebiete der Röntgenstrahlen. Bd. iv., Heft i., June, 1901, and Archives, Aug. [8] Amer. Jour. Med. Science, Nov., 1901. [9] Brit. Med. Jour., Aug. 24, 1901. [10] Lancet, Sep. 21, 1901. [11] Archives of the Roentgen Ray, Aug., 1901.

